# Roll to roll in situ preparation of recyclable, washable, antibacterial Ag loaded nonwoven fabric

**DOI:** 10.1038/s41598-022-17484-6

**Published:** 2022-08-01

**Authors:** Yanfang Xu, Lulu Tian, Junfang Li, Xiaohui Lv, Fei Li, Li Sun, Liyong Niu, Xiaohong Li, Zhijun Zhang

**Affiliations:** 1 Engineering Research Center for Nanomaterials, Henan University, Kaifeng, 475004 China; 2 Engineering Research Center for Nanomaterials Co. Ltd., Henan University, Jiyuan, 459000 China; 3 College of Chemistry and Chemical Engineering, Henan University, Kaifeng, 475004 China

**Keywords:** Health care, Materials science, Nanoscience and technology

## Abstract

Functional fabrics with antibacterial performance are more welcome nowadays. However, the fabrication of functional fabrics with durable, steady performance via a cost-effective way remains a challenge. Polypropylene (denoted as PP) nonwoven fabric was modified by polyvinyl alcohol (denoted as PVA), followed by the in-situ deposition of silver nanoparticles (denoted as Ag NPs) to afford PVA-modified and Ag NPs-loaded PP (denoted as Ag/PVA/PP) fabric. The encapsulation of PP fiber by PVA coating contributes to greatly enhancing the adhesion of the loaded Ag NPs to the PP fiber, and the Ag/PVA/PP nonwoven fabrics exhibit significantly improved mechanical properties as well as excellent antibacterial activity against *Escherichia coli* (coded as *E. coli*). Typically, the Ag/PVA/PP nonwoven fabric obtained at a silver ammonia concentration of 30 mM has the best mechanical properties and the antibacterial rate reaches 99.99% against *E. coli*. The fabric retains excellent antibacterial activity even after washing for 40 cycles, showing prospects in reuse. Moreover, the Ag/PVA/PP nonwoven fabric could find promising application in industry, thanks to its desired air-permeability and moisture-permeability. In addition, we developed a roll-to-roll production process and conducted preliminary exploration to verify the feasibility of this method.

## Introduction

Mass population movements along with the deepening of economic globalization have greatly increased the possibility of virus spreading, which could well explain why it is difficult to prevent the epidemic of new corona virus with strong ability to spread worldwide^1–3^. In this sense, it is urgent to develop novel antibacterial materials like polypropylene (PP) nonwoven fabrics as medical protective materials. PP nonwoven fabrics exhibit low density, chemical inertness, low-cost and other advantages^4^, they alone, unfortunately, have no antibacterial capability and exhibit short service life and low protection efficiency. Therefore, it is of significance to endow PP nonwoven fabric with antibacterial ability.

Silver, an ancient antibacterial agent, has undergone five stages of development, including colloidal silver solution, sulfadiazine silver, silver salt, protein silver and nano silver. The application of nano-silver is more and more extensive, such as medical field^5,6^, electrical conductivity^7–9^, surface-enhanced Raman^10–12^, catalytic degradation of dyes^13–16^ and so on. Especially, silver nanoparticles (Ag NPs) are advantageous over conventional antimicrobial agents such as metal salt, quaternary ammonium compound and triclosan, thanks to their desired bacterial resistance, stability, low-cost and environmental acceptance^17–19^. Besides, Ag NPs with large specific surface area and high antibacterial activity can be attached to fabrics including wool fabrics^20^, cotton fabrics^21,22^, and polyester fabrics to achieve continuous release of antibacterial silver particles in a controllable mode^23,24^. This means it could be feasible to fabricate PP fabrics possessing antibacterial activity by encapsulation with Ag NPs. However, PP nonwoven fabrics are lack of functional groups and exhibit low polarity^25^, which is unfavorable for their encapsulation by Ag NPs. To overcome this drawback, some researchers have attempted to adopt various modification methods including plasma sputtering^26,27^, radiation grafting^28–31^, and surface coating^32^ to deposit Ag NPs on PP fabrics surface. For example, Goli et al.^33^ introduced a protein coating on the surface of PP nonwoven fabric; the amino acid on the periphery of the protein layer could act as anchoring points for the combination of Ag NPs, thereby achieving good antibacterial activity. Li and co-workers^34^ found that *N*-isopropylacrylamide and *N*-(3-aminopropyl) methacrylamidehydrochchloride co-grafted via ultraviolet (UV) etching exhibited efficient antibacterial activity, although the UV etching process was complicated and might worsen the mechanical properties of the fibers. By pre-treating pristine PP under gamma radiation, Oliani et al^35^ fabricated Ag NPs-PP gel films with outstanding antibacterial activity; their approach, however, was also complicated. In summary, it still remains a challenge to prepare recyclable PP nonwoven fabrics with desired antibacterial activity in an efficient and facile manner.

In the present research, polyvinyl alcohol, a green and cheap membrane material with good film-forming ability, high hydrophilicity, and outstanding physicochemical stability, is used to modify PP fabric. And glucose is used as reducing agent^36^. The increased surface energy of modified PP is favorable for the selective deposition of Ag NPs. Compared with pure PP fabric, the as-prepared Ag/PVA/PP fabrics exhibit good recyclability, excellent antibacterial activity against *Escherichia coli*, good mechanical properties even after washing for 40 cycles as well as comparable air- and moisture-permeability.

## Experimental

### Materials

PP nonwoven fabric with a unit weight of 25 g/m^2^ and a thickness of 0.18 mm was kindly supplied by Jiyuan Kangan Sanitary Materials Company Limited (Jiyuan, China) and cut into plates with a size of 5 × 5 cm^2^. Silver nitrate (99.8%; AR) was obtained from Xilong Scientific Company Limited (Shantou, China). Glucose was purchased from Fuzhou Haiwangxing Fuyao Pharmaceuticals Company Limited (Fuzhou, China). Polyvinyl alcohol (industrial grade reagent) was received from Tianjin Sitong Chemical Plant (Tianjin, China). Deionized water was used as a solvent or for rinsing and prepared at our laboratory. Nutrient agar and broth were obtained from Beijing Aoboxing Biotechnology Corporation (Beijing, China). *E. coli* (ATCC 25922) bacterial strains were purchased from Zhangzhou Bochuang Corporation (Zhangzhou, China).

### Synthesis

#### Preparation of PVA/PP composite fabric

The as-received PP fabric was ultrasonically rinsed with ethanol for 15 min. The as-received PVA was added into water and heated at 95 °C for 2 h to obtain the aqueous solution. Then glucose was dissolved in 10 mL of the PVA solution at a mass fraction of 0.1%, 0.5%, 1.0%, and 1.5%. The as-cleaned PP nonwoven fabric was dipped into the PVA/glucose solution and heated at 60 °C for 1 h. Upon completion of heating, the as-dipped PP nonwoven fabric was lifted from the PVA/glucose solution and dried at 60 °C for 0.5 h to form PVA film on the fabric surface, thereby affording the PVA/PP composite fabric.

#### Preparation of Ag/PVA/PP composite fabric

Silver nitrate was dissolved in 10 mL of water on the condition of stirring constantly at room temperature, followed by the dropwise addition of ammonia until the solution changed from clear to brownish yellow and clear again to obtain silver ammonia solution (5–90 mM). The PVA/PP nonwoven fabric was put into the silver ammonia solution and heated at 60 °C for 1 h to allow the in-situ generation of Ag NPs on the fabric surface, followed by rinsing three times with water and drying at 60 °C for 0.5 h to obtain Ag/PVA/PP composite fabrics.

After preliminary experiments, we have set up a roll-to-roll equipment in the laboratory for larger-scale production of composite fabric. The rollers are made of Teflon to avoid side reaction and any contaminations. In this process, we can adjust the rolling speed and roller spacing to control the dipping time and adsorption amount of solution so as to obtain desired Ag/PVA/PP composite fabric.

### Characterizations

The surface morphology of the fabrics was investigated with a VEGA3 scanning electron microscope (SEM; Japan Electronics, Japan) at the accelerating voltage of 5 kV. X-ray diffraction in a 2*θ* range of 10°–80° (XRD; Bruker, D8 Advanced, Germany; Cu *K*α radiation, *λ* = 0.15418 nm; voltage: 40 kV, current: 40 mA) was used to analyzed the crystal structure of Ag NPs. A Fourier transform infrared spectrometer (ATR-FTIR; Nicolet 170sx, Thermo Fisher Scientific Incorporation) was employed to analyze the chemical features of the PP fabric after surface modification. The PVA modifier content of Ag/PVA/PP composite fabric was measured by thermogravimetric analysis (TGA; Mettler Toledo, Switzerland) in flowing nitrogen gas. The silver content of Ag/PVA/PP composite fabrics was detected by the inductively coupled plasma mass spectrometry (ICP-MS, ELAN DRC II, Perkin-Elmer (Hong Kong) Company Limited).

### Determination of air permeability and water vapor transmission rate

The air permeability and water vapor transmission rate of Ag/PVA/PP composite fabrics (size: 78 × 50 cm^2^) were determined by a third-party testing agency (TianFangBiao Standardization certification & Testing Co., Ltd) according to GB/T 5453-1997 and GB/T 12704.2-2009. Ten different points of each sample are chosen for testing, and the data provided by the agency is the average value of the ten points.

#### Evaluation of antibacterial activity of Ag/PVA/PP composite fabric

The antibacterial activity of Ag/PVA/PP composite fabric was measured by agar plate diffusion method (for qualitative analysis) and flask-shaking method (for quantitative analysis), according to Chinese standards GB/T 20944.1-2007 and GB/T 20944.3-2008, respectively. The antibacterial rate of Ag/PVA/PP composite fabrics against *E. coli* upon washing for different duration was determined. As to the agar plate diffusion method, the to-be-tested Ag/PVA/PP composite fabric was made into a disc (diameter: 8 mm) with a puncher and attached to the agar petri dish inoculated with E. coli (ATCC 25922; 3.4 × 10^8^ CFU mL^−1^), followed by incubation at 37 °C and a relative humidity of 56% for about 24 h. The inhibition zone was analyzed perpendicularly from the center of the disc to the inner periphery of the bacterial colonies around. For the flask-shaking method, the to-be-tested Ag/PVA/PP composite fabric was made into a plate with a size of 2 × 2 cm^2^ and autoclaved in a broth medium at 121 °C and 0.1 MPa for 30 min. Upon completion of autoclaving, the samples were dipped into an erlenmeyer flask containing 5 mL of bacterial suspension in 70 mL of broth culture solution (the concentration of the suspensions is 1 × 10^5^–4 × 10^5^ CFU/mL), followed by shaking at 150 rev/min and 25 °C for 18 h. At the end of shaking, a certain amount of the bacterial suspension was collected and diluted by tenfold. An appropriate amount of the as-diluted bacterial suspension was collected and spread onto the agar medium, followed by incubation at 37 °C and a relative humidity of 56% for 24 h. The antibacterial rate is calculated as $\frac{\mathrm{C}-\mathrm{A}}{\mathrm{C}}\cdot 100\%$, where *C* and *A* are the numbers of colonies after 24 h culture in the blank control group and the Ag/PVA/PP composite fabrics, respectively.

### Washing test

The durability of the Ag/PVA/PP composite fabrics was evaluated by washing in accordance with the standard ISO 105-C10:2006.1A. During washing, the to-be-tested Ag/PVA/PP composite fabrics (30 × 40 mm^2^) were immersed in an aqueous solution containing commercial detergent (5.0 g/L) and stirred at 40 ± 2 rev/min and 40 ± 5 °C for 10, 20, 30, 40, and 50 cycles. After laundering, the fabrics were rinsed three times with water and dried at 50–60 °C for 30 min. The changes of silver content after washing were determined to afford bacteriostatic rate.

## Results and discussion

### Characterization of the composite fabric

**Figure 1:**
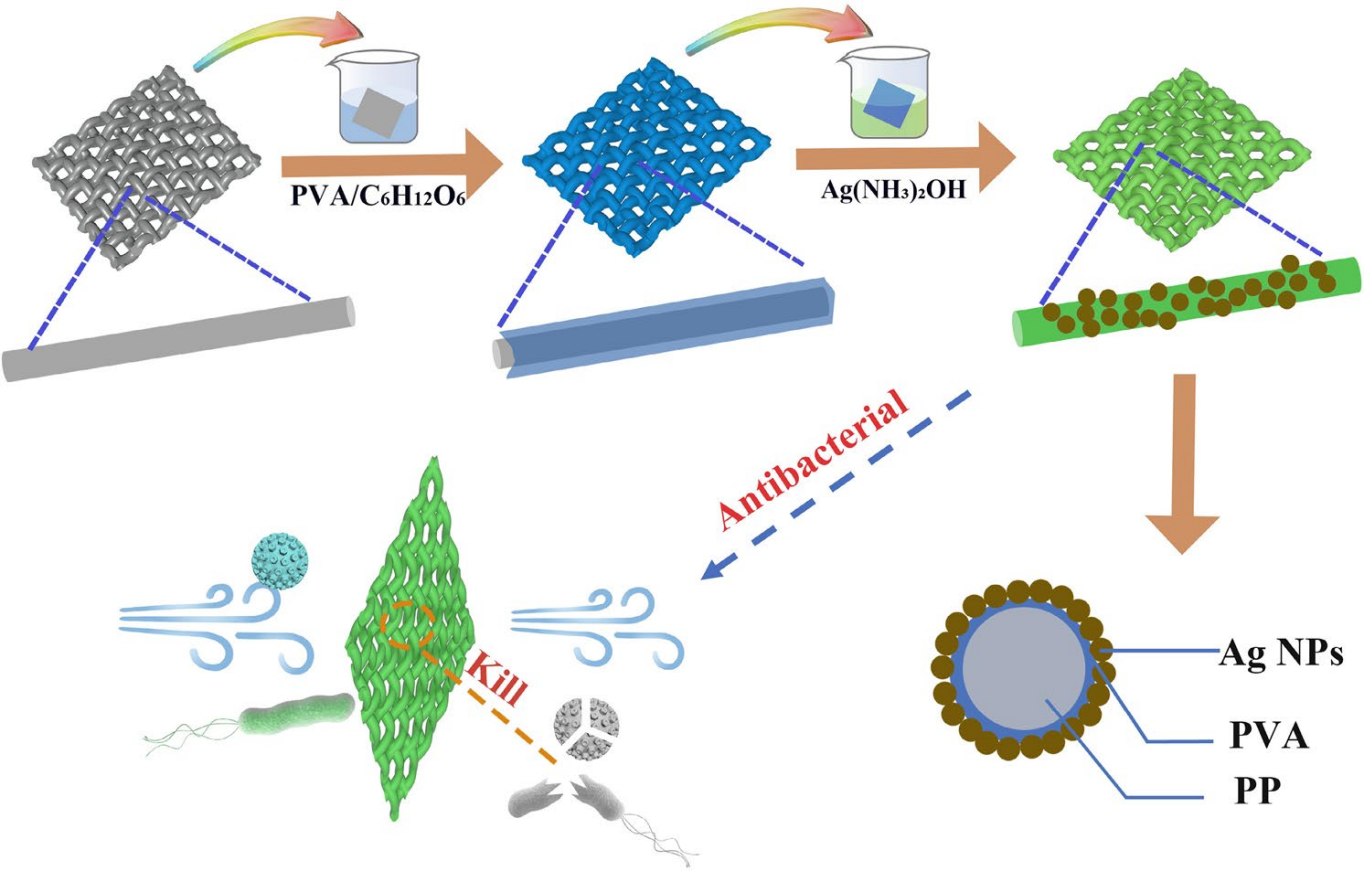
Schematic diagram showing the preparation of Ag/PVA/PP fabrics and their antibacterial action.

**Figure 2:**
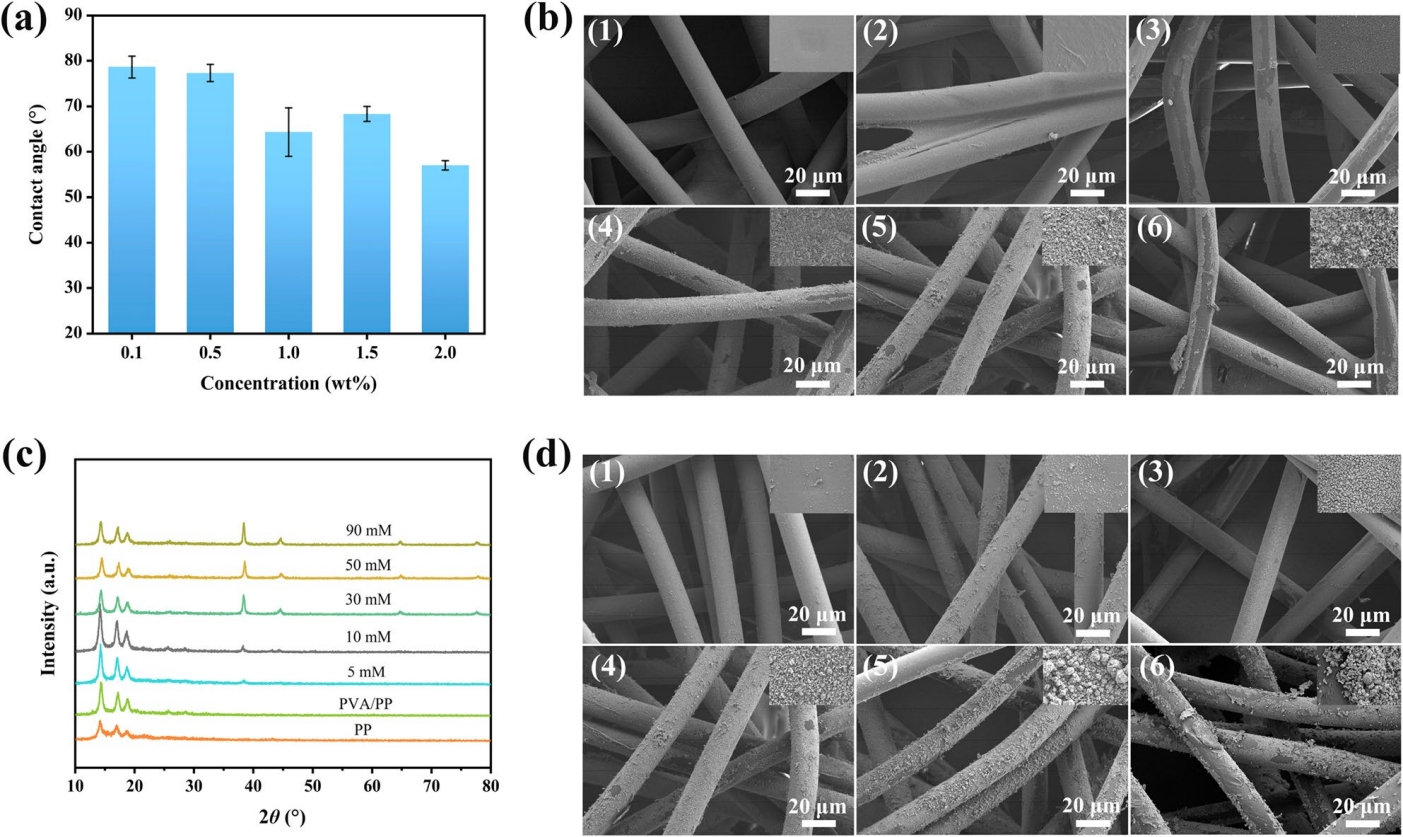
Variation of water contact angle of Ag/PVA/PP fabrics with different PVA concentrations (**a**), SEM images of Ag/PVA/PP fabrics obtained at the silver ammonia concentration of 50 mM and different molar ratios of glucose and silver ammonia [(**b**); (**1**) PP fiber, (**2**) PVA/PP fiber, (**3**) molar ration 1:1, (**4**) molar ratio 3:1, (**5**) molar ratio 5:1, and (**6**) molar ratio 9:1], XRD patterns (**c**) and SEM images (**d**) of Ag/PVA/PP fabrics obtained under silver ammonia concentrations of (**1**) 5 mM, (**2**) 10 mM, (**3**) 30 mM, (**4**) 50 mM, (**5**) 90 mM, and (**6**) Ag/PP-30 mM. The reaction temperature is 60 °C.

Figure 1 shows the schematic diagram for preparing Ag/PVA/PP composite fabrics. Namely, the PP nonwoven fabric is immersed in the mixed solution of PVA and glucose. The as-immersed PP nonwoven fabric is dried to fix the modifier and reducing agent, thereby forming the encapsulation layer. The as-dried PP nonwoven fabric is immersed in silver ammonia solution to achieve in-situ deposition of Ag NPs, where the concentration of the modifier, the molar ratio of glucose and silver ammonia, the concentration of silver ammonia, and the reaction temperature are important factors influencing the deposition of Ag NPs. Figure 2a exhibits the relationship between the water contact angle of Ag/PVA/PP fabrics and the concentration of the modifier. When the concentration of the modifier increases from 0.5 to 1.0 wt%, the contact angle of Ag/PVA/PP fabrics tends to decrease obviously; and it remains nearly unchanged when the concentration of the modifier increases from 1.0 to 2.0 wt%. Figure 2b shows the SEM images of pure PP fiber and of Ag/PVA/PP fabrics obtained at silver ammonia concentration of 50 mM and different molar ratios of glucose and silver ammonia (1:1, 3:1, 5:1 and 9: ). The as-received PP fiber is relatively smooth. After the encapsulation by PVA film, some fibers are bonded together; and the fibers become relatively rough, due to the deposition of Ag NPs. The deposition layer of Ag NPs gradually becomes thicker as the molar ratio of reducing agent and glucose increases; and Ag NPs tend to form aggregates when the molar ratio increases to 5:1, and 9:1. Particularly, when the molar ratio of reducing agent and glucose is 5:1, the macroscopic and microscopic images of the PP fibers are relatively uniform. The digital photos of corresponding samples obtained at silver ammonia concentration of 50 mM are shown in Fig. S1.

Figure 2c shows the XRD patterns of the as-obtained Ag/PVA/PP fabrics. Aside from the diffraction peaks of PP fiber^37^, the four diffraction peaks at 2*θ* = ∼ 37.8°, 44.2°, 64.1°, and 77.3° correspond to the (1 1 1), (2 0 0), (2 2 0), and (3 1 1) crystal planes of face-centered-cubic Ag NPs. As the silver ammonia concentration increases from 5 to 90 mM, the XRD peaks of Ag become sharper, corresponding to increased crystallinity therewith. According to the Scherrer formula, the grain size of the Ag NPs obtained with 10 mM, 30 mM, and 50 mM silver ammonia is calculated to be 21.3 nm, 23.3 nm, and 26.5 nm, respectively. This is because the concentration of silver ammonia is the driving force of the reducing reaction to afford metallic Ag. And the nucleation and growth rate of Ag NPs increases with increasing the concentration of silver ammonia. Figure 2d shows the SEM images of Ag/PVA/PP fabrics obtained at different silver ammonia concentrations. When the silver ammonia concentration is 30 mM, the deposition layer of Ag NPs is relatively uniform. However, the uniformity of the Ag NPs deposition layer tends to decrase when the silver ammonia concentration is too high, possibly due to serious agglomeration of Ag NPs thereat. Besides, there are two shapes of nano-silver on the surface: spherical and flake. The spherical particle size is about 20–80 nm, and the lateral size of the flake is about 100–300 nm (Fig. S2). The deposition layer of Ag NPs on the surface of unmodified PP fabric is uneven. Furthermore, elevating temperature contributes to promoting the reduction of Ag NPs (Fig. S3), but too high reaction temperature is unfavorable for the selective deposition of Ag NPs.

**Figure 3:**
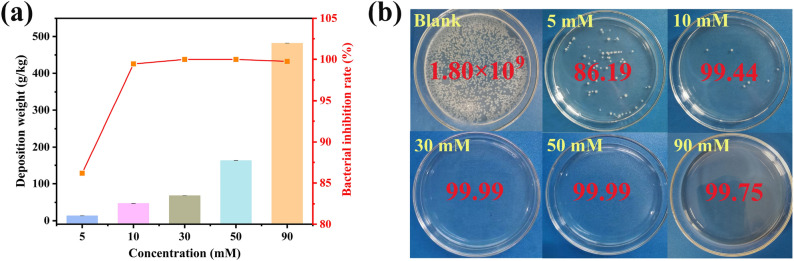
(**a**) Relationship among antibacterial rate and amount of Ag deposition layer as well as concentration of silver ammonia; (**b**) Digital camera pictures of bacterial culture plates showing antibacterial activity of blank sample and Ag/PVA/PP fabrics obtained with 5 mM, 10 mM, 30 mM, 50 mM, and 90 mM of silver ammonia against *E. coli.*

Figure 3a schematically describes the relationship between silver ammonia concentration, silver deposition amount and antibacterial rate of as-prepared Ag/PVA/PP fabrics. Figure 3b shows the antibacterial pictures of the samples under different silver ammonia concentrations, which can directly reflect the antibacterial situation of the samples. When the concentration of silver ammonia increases from 5 to 90 mM, the silver deposition amount increases from 13.67 to 481.81 g/kg. Besides, the antibacterial rate against *E. coli* initially increases and then remains at a high level with the increase of silver deposition amount. Particularly, when the concentration of silver ammonia is 30 mM, the as-obtained Ag/PVA/PP fabric has a silver deposition amount of 67.62 g/kg and an antibacterial rate of 99.99%. And this sample is selected as the representative for subsequent structural characterization.

**Figure 4:**
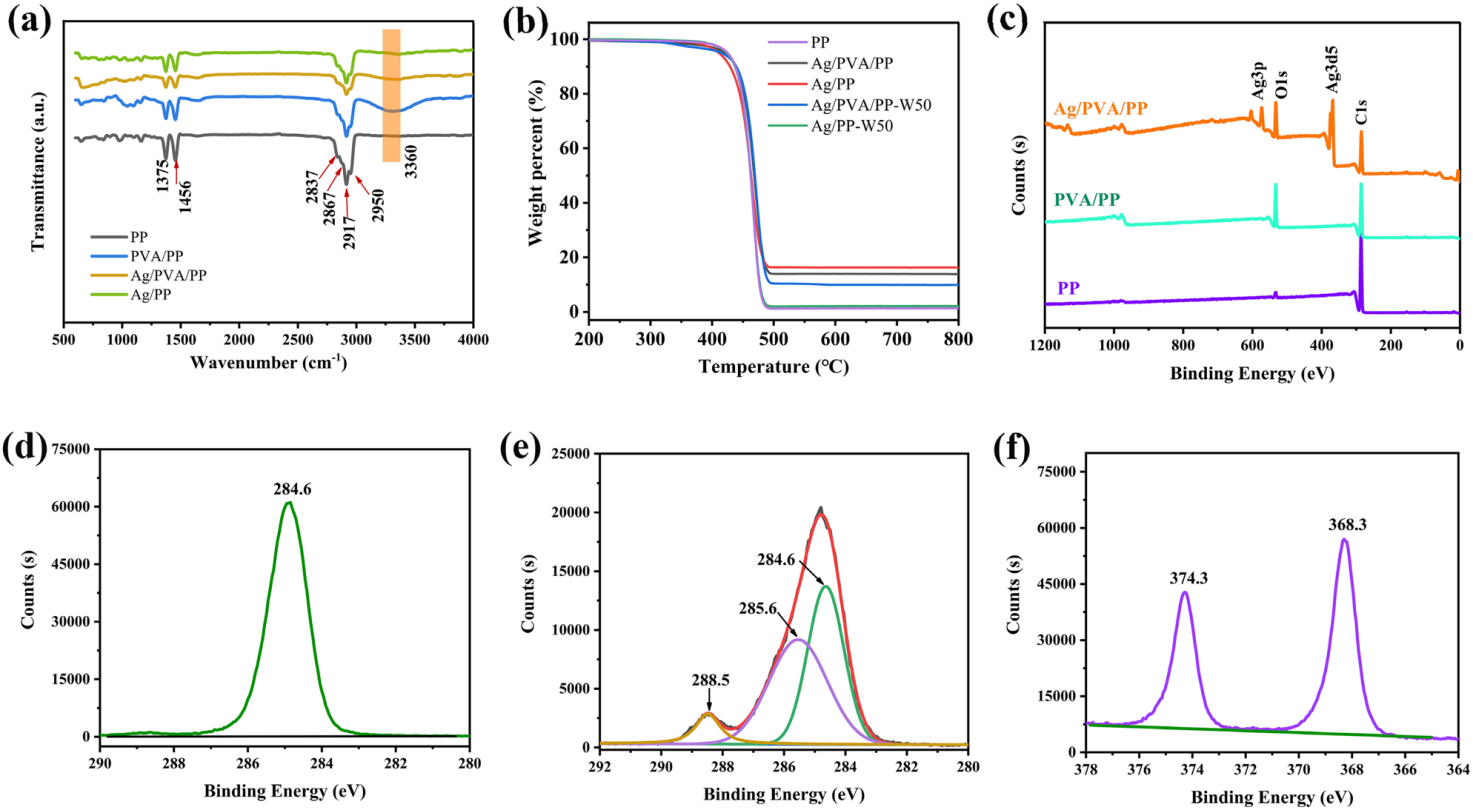
FTIR spectra (**a**), TGA curves (**b**), and XPS survey spectra (**c**) of pure PP, PVA/PP fabric, and Ag/PVA/PP fabric as well as C 1*s* spectra of pure PP (**d**), PVA/PP fabric (**e**), and Ag 3*d* peak of Ag/PVA/PP fabric (**f**).

Figure 4a shows the FT-IR/ATR spectra of PP, PVA/PP, Ag/PP, and Ag/PVA/PP. The absorbance bands of pure PP fiber at 2950 cm^−1^ and 2916 cm^−1^ are due to the asymmetric stretching vibration of –CH_3_ and –CH_2_– groups; and those at 2867 cm^−1^ and 2837 cm^−1^ are assigned to the symmetric stretching of –CH_3_ and –CH_2_–. The absorbance bands at 1375 cm^−1^ and 1456 cm^−1^ are ascribed to the asymmetric and symmetric scissoring vibrations of –CH_3_^38,39^. The FTIR spectrum of Ag/PP fiber is similar to that of PP fiber. Aside from the absorbance bands of PP, the new absorbance peak of PVA/PP and Ag/PVA/PP fabrics at 3360 cm^−1^ is assigned to the stretch of the hydrogen bond of –OH group. This indicates that PVA is successfully coated onto the surface of PP fiber. Furthermore, the hydroxyl absorbance peak of Ag/PVA/PP fabric is slightly weaker than that of PVA/PP, which is possibly because a part of the hydroxyl group is coordinated with silver^40^.

Figure 4c presents the XPS survey spectra of PP, PVA/PP and Ag/PVA/PP fabrics. The weak O 1*s* signal of neat PP fiber could be assigned to oxygen element adsorbed on the surface; and the C 1*s* peak at 284.6 eV is attributed to C–H and C–C (see Fig. 4d). As compared with neat PP fiber, PVA/PP fabric (Fig. 4e) shows strong O 1*s* signal as well as C 1*s* signals at 284.6 eV (C–C/C–H), 285.6 eV (C–O–H), and 288.5 eV (H–C=O)^38^. Besides, the O 1*s* spectrum of PVA/PP fabric can be fitted into two peaks at 532.3 eV and 533.2 eV^41^ (Fig. S4); and these C 1*s* peaks correspond to C–OH and H–C=O (the hydroxyl group of PVA and the aldehyde group of glucose), respectively, which is consistent with the FTIR data. The Ag/PVA/PP nonwoven fabric retains the O 1*s* spectrum (Fig. S5) of C–OH (532.3 eV) and H–C=O (533.2 eV), and it consists of 65.81% (atomic percentage) C, 22.89% O, and 11.31% Ag (Fig. S4). Particularly, the Ag 3*d*_5/2_ and Ag 3*d*_3/2_ peaks at 368.2 eV and 374.2 eV (Fig. 4f) further proves that Ag NPs are doped on the surface of the PVA/PP nonwoven fabric^42^.

The TGA curves of pure PP, Ag/PP fabric and Ag/PVA/PP fabric (Fig. 4b) indicate that they undergo similar thermal decomposition process, and the deposition of Ag NPs leads to slight increase in the thermal degradation temperature of PP fiber and PVA/PP fiber (from 480 °C (PP fiber) to 495 °C), possibly due to the formation of the Ag barrier layer^43^. Besides, the residues of pure PP, Ag/PP, Ag/PVA/PP, Ag/PVA/PP-W50, and Ag/PP-W50 samples after being heated at 800 °C are 1.32%, 16.26%, 13.86%, 9.88% and 2.12%, respectively (here suffix W50 refers to 50 cycles of washing). The residue of pure PP is attributed to the impurities, and those of the other samples are attributed to Ag NPs; and the difference in the residue amounts of the Ag-loaded samples should be due to the difference in the amounts of their loaded Ag NPs. Moreover, the remaining amount of silver decreases by 94.65% after Ag/PP fabric is washed for 50 cycles, and that of the Ag/PVA/PP fabric decreases by about 31.74%. This indicates that the PVA encapsulation coating can effectively enhance the adhesion of Ag NPs to PP matrix.

### Comfort and mechanical properties

**Figure 5:**
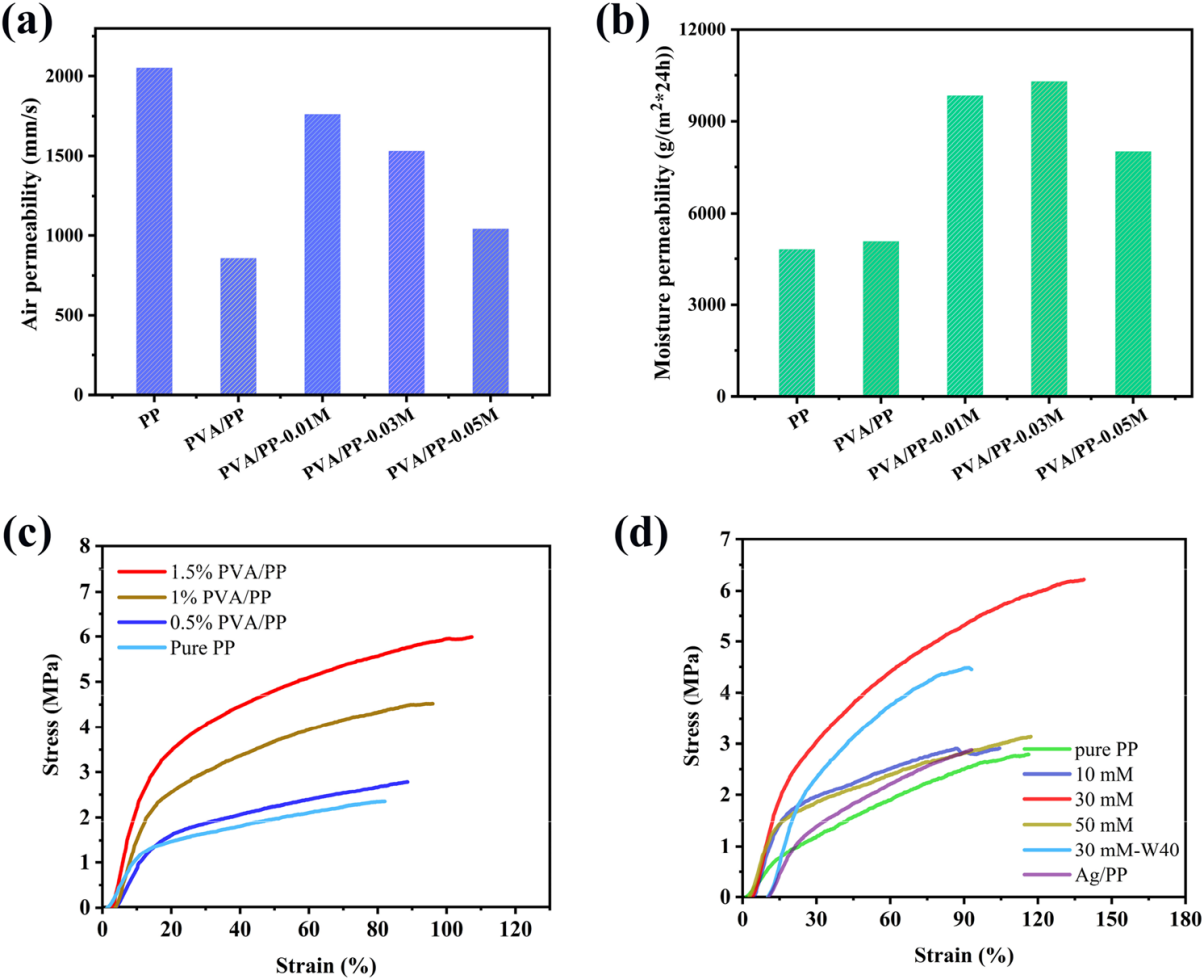
(**a**) Air permeability of Ag/PVA/PP fabrics obtained under different silver ammonia concentrations; (**b**) Water vapor transmission rate of Ag/PVA/PP fabrics obtained under different silver ammonia concentrations; (**c**) Tensile curves of Ag/PVA/PP fabrics obtained under different modifier concentrations; (**d**) Tensile curves of Ag/PVA/PP fabrics obtained under different silver ammonia concentrations (that of the Ag/PVA/PP fabric obtained under the silver ammonia concentration of 30 mM after being washed for 40 cycles is also given for a comparison).

The air permeability and water vapor transmission rate of as-prepared PP fabrics were measured to estimate their comfort for wearing. Generally, air permeability is associated with the thermal comfort of the wearer, especially in hot and humid environments^44^. As shown in Fig. 5a, the air permeability of pure PP is 2050 mm/s, and it decreases to 856 mm/s after the modification by PVA. This is because the PVA film formed on the surface and the interlaced part of the PP fiber contributes to reducing the gap between the fibers. After the deposition of Ag NPs, the air permeability of the PP fabrics increases, due to the consumption of the PVA coating during the deposition process of Ag NPs. Besides, the air permeability of the Ag/PVA/PP fabrics tends to decrease with increasing silver ammonia concentration from 10 to 50 mM. This is possibly because the thickness of the silver deposition layer increases with increasing the concentration of silver ammonia, which contributes to reducing the amount of pores as well as the chance for water vapor to pass through the pores.

Water vapor transmission rate is another important indicator to measure the thermal comfort of fabrics^45^. It has been proven that the moisture permeability of fabrics is mainly affected by air permeability and surface properties. Namely, the air permeability mainly depends on the number of pores; and the surface properties affect the moisture permeability of hydrophilic group via adsorption–diffusion–desorption of water molecules. As shown in Fig. 5b, the moisture permeability of pure PP fiber is 4810 g/(m^2^·24 h). After the encapsulation by PVA coating, the amount of holes of PP fiber decreases; but the moisture permeability of PVA/PP fabric increases to 5070 g/(m^2^·24 h), which is because its moisture permeability is dominated by surface properties rather than the pores. After the deposition of Ag NPs, the moisture permeability of Ag/PVA/PP fabrics further increases. Particularly, the Ag/PVA/PP fabric obtained at a silver ammonia concentration of 30 mM has the maximum moisture permeability of 10,300 g/(m^2^·24 h). Here the different moisture permeability of Ag/PVA/PP fabrics obtained under different silver ammonia concentration could be related to the difference in the thickness of the silver deposition layer as well as the amount of the pores thereat.

The mechanical properties of fabric seriously affect its service life, especially as a recyclable material^46^. Figure 5c shows the stress–strain curves of the Ag/PVA/PP fabrics. The tensile strength of pure PP is only 2.23 MPa, and that of 1 wt% PVA/PP fabric significantly increases to 4.56 MPa, which indicates that the encapsulation of PP fabric by PVA contributes to greatly increasing the mechanical properties. The tensile strength and elongation at break of PVA/PP fabrics increase with increasing the concentration of PVA modifier, which is because the PVA film can share stress and function to strengthen and toughen PP fiber. However, when the concentration of the modifier increases to 1.5 wt%, the viscous PVA makes the PP fabric hard, thereby seriously worsening the comfort for wearing.

As compared with pure PP and PVA/PP fabrics, Ag/PVA/PP fabrics exhibit further enhanced tensile strength and elongation at break, which is because the Ag NPs uniformly distributed on the surface of the PP fiber can share the load^47,48^. It can be seen the tensile strength of Ag/PP fiber is higher than pure PP and reaches to 3.36 MPa (Fig. 5d), which confirms the strengthening and toughening effect of Ag NPs. Particularly, the Ag/PVA/PP fabric obtained under the silver ammonia concentration of 30 mM (rather than 50 mM) exhibits the maximum tensile strength and elongation at break, which is still due to the uniform deposition of Ag NPs thereat as well as the aggregation of Ag NPs under a high concentration of silver ammonia. Moreover, after being washed for 40 cycles, the tensile strength and elongation at break of the Ag/PVA/PP fabric obtained under silver ammonia concentration of 30 mM are reduced by 32.7% and 26.8% (Fig. 5d), respectively, possibly due to the slightly loss of the deposited Ag NPs thereafter.

### Washable stability

**Figure 6:**
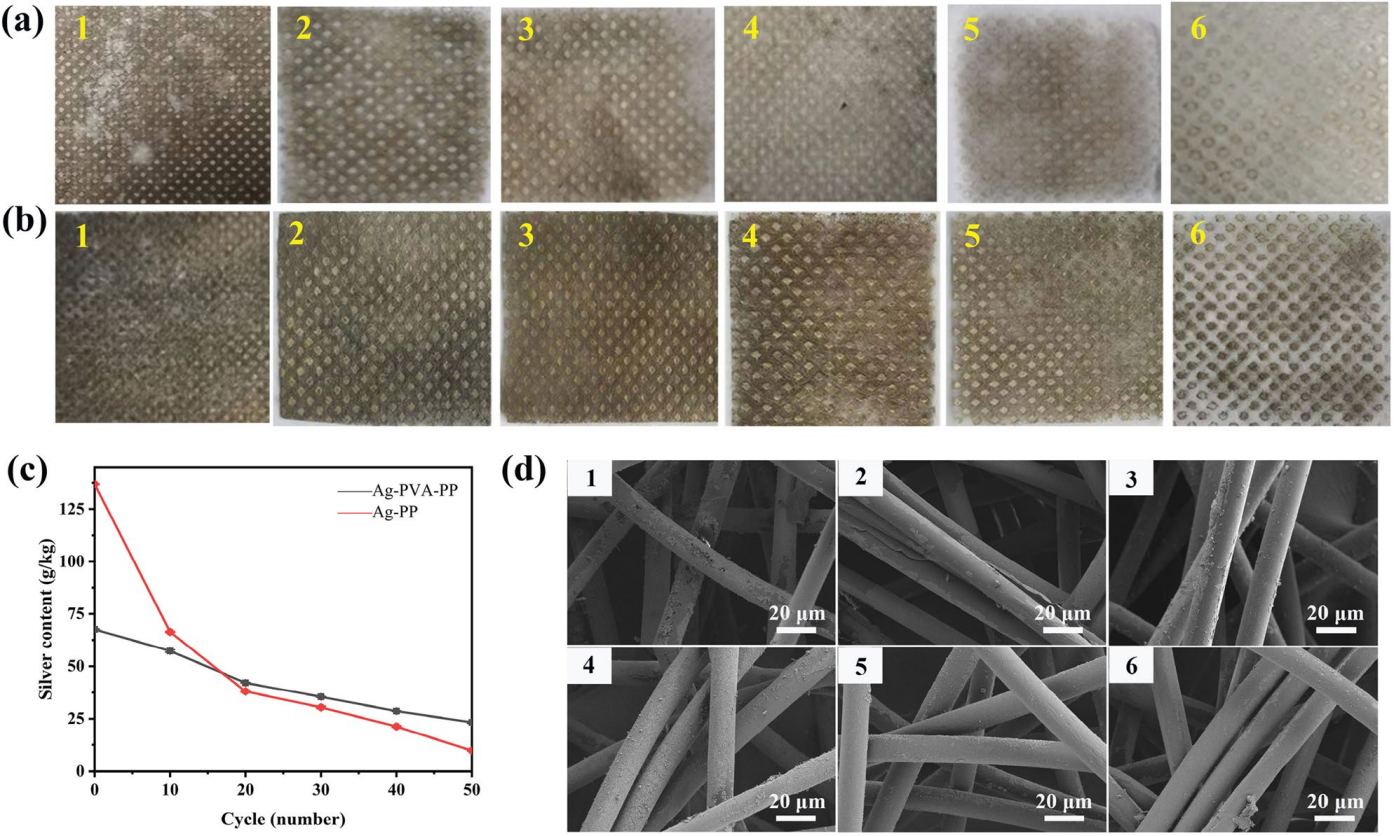
(**a**) Digital camera pictures of Ag/PP fabric (obtained under the silver ammonia concentration of 30 mM) after washing for 0, 10, 20, 30, 40, and 50 cycles (1–6); (**b**) Digital camera pictures of Ag/PVA/PP fabric (obtained under the silver ammonia concentration of 30 mM) after washing for 0, 10, 20, 30, 40, and 50 cycles (1–6); (**c**) silver content changes on two fabrics with washing cycles; and (**d**) SEM images of the Ag/PVA/PP fabric (1–3) and Ag/PP fabric (4–6) after washing for 10, 40, and 50 cycles.

Figure 6a and b shows the digital camera pictures of Ag/PVA/PP fabric and Ag/PP fabric obtained under the silver ammonia concentration of 30 mM after washing for 0, 10, 20, 30, 40, and 50 cycles. The dark-grey Ag/PVA/PP fabric and Ag/PP fabric gradually become light-grey after washing; and it seems that the former undergoes less severe change of color upon washing than the latter. Besides, compared to Ag/PP fabric, the silver content of Ag/PVA/PP fabric declines relatively slowly after washing; and after washing 20 cycles and even more, the former retains a higher silver content than the latter (Fig. 6c). This indicates that the encapsulation of PP fiber by PVA coating contributes to significantly improving the adhesion of Ag NPs to the PP fiber. Figure 6d shows the SEM images of Ag/PVA/PP fabric and Ag/PP fabric after washing for 10, 40, and 50 cycles. Ag/PVA/PP fabric undergoes less loss of Ag NPs than Ag/PP fabric upon washing, which is still because the PVA encapsulation coating contributes to enhancing the adhesion of Ag NPs to the PP fiber.

**Figure 7:**
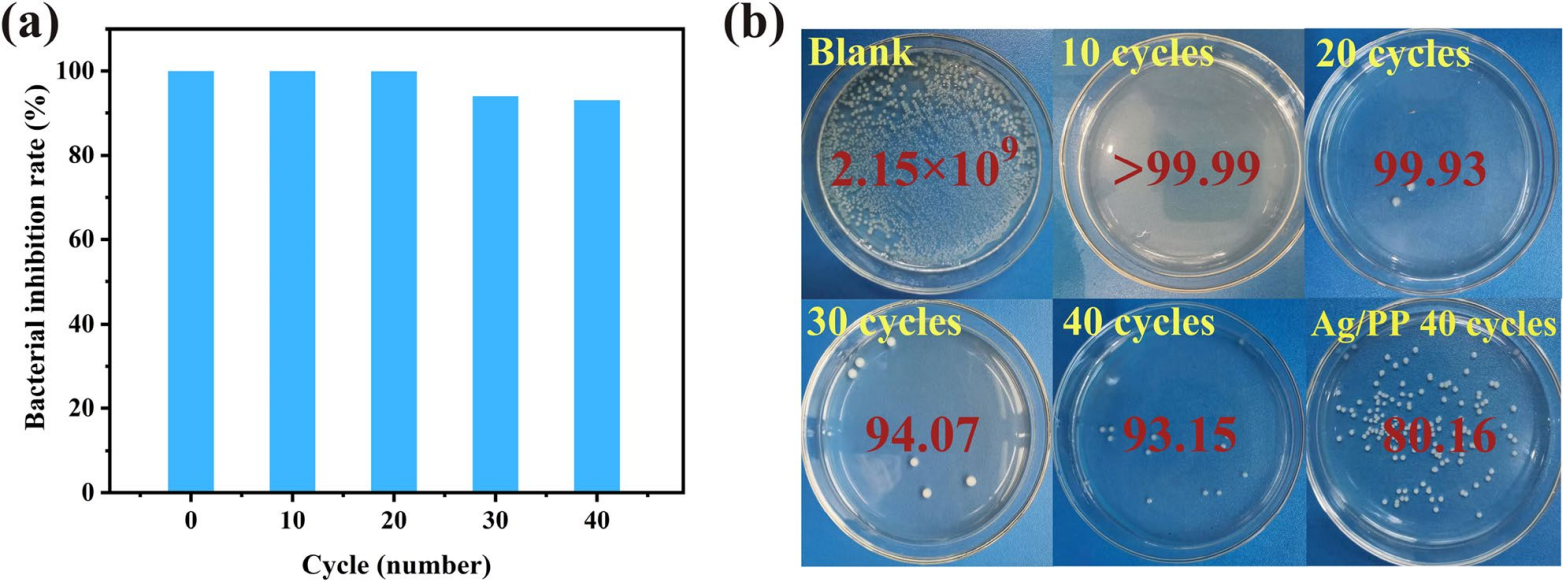
(**a**) Antibacterial rate against *E. coli*. (**b**) Digital camera pictures of Ag/PVA/PP fabric after being washed for 10, 20, 30, 40 cycles, and 40 cycles of the Ag/PP fabric obtained under the silver ammonia concentration of 30 mM is also given for a comparison.

Figure 7 shows the antibacterial rate and digital camera pictures of Ag/PVA/PP fabric against *E. coli* after being washed for 10, 20, 30, and 40 cycles. After being washed for 10 and 20 cycles, the Ag/PVA/PP fabric retains an antibacterial rate of 99.99% and 99.93%, showing excellent antibacterial activity. And the antibacterial rate of the Ag/PVA/PP fabric decreases slightly after washing for 30 and 40 cycles, due to the loss of Ag NPs upon washing for extended cycles. However, the antibacterial rate of the Ag/ PP fabric after washing for 40 cycles is only 80.16%. Obviously, the antibacterial effect of Ag/PP fabric is far less than that of Ag/PVA/PP after washing for 40 cycles.

### Roll-to-roll process design

**Figure 8:**
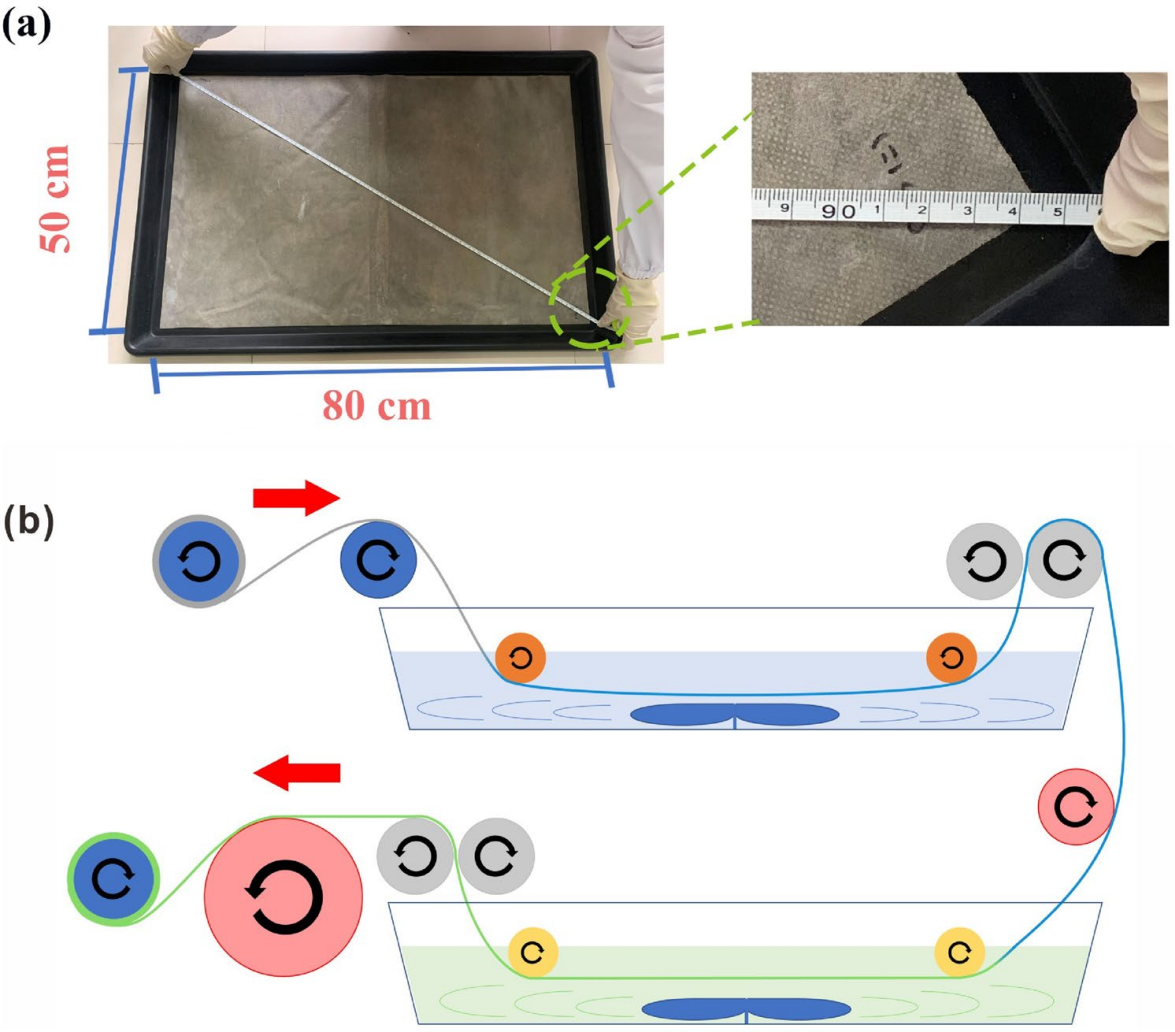
Schematic diagrams showing the fabrication of large-size target product (**a**) and the roll-to-roll production process of Ag/PVA/PP nonwoven fabric (**b**).

Figure 8 schematically shows the preparation of a large-size Ag/PVA/PP fabric via a two-step roll-to-roll route. Namely, the PVA/glucose solution is immersed in the roll-to-roll frame for a certain period of time and taken out, followed by the impregnation of silver ammonia solution in the same manner affording Ag/PVA/PP fabric (Fig. 8a). The as-obtained Ag/PVA/PP fabric still retains excellent antibacterial activity even after being placed for 1 year. As to the large-scale preparation of Ag/PVA/PP fabric, the as-received nonwoven PP fabric is impregnated in a continuous roll-to-roll manner, followed by passing through PVA/glucose solution and silver ammonia solution in turn with two connected rollers. The dipping time is controlled by adjusting the roller speed and the adsorption amount of the solutions is controlled by adjusting the roller spacing (Fig. 8b), thereby affording the target large-size Ag/PVA/PP nonwoven fabric (50 cm × 80 cm) with the collecting roller. The whole process is simple and efficient, which could be favorable for large-scale production.

## Conclusions

Silver-loaded PVA/PP nonwoven fabric is prepared by a facile liquid phase in-situ deposition technology in association with roll-to-roll route. The as-prepared Ag/PVA/PP nonwoven fabric exhibits greatly improved mechanical properties as compared with PP fabric and PVA/PP fabric, which is because the PVA encapsulation layer can significantly enhance the adhesion of Ag NPs to the PP fiber. Besides, the loaded PVA amount and the Ag NPs content of Ag/PVA/PP nonwoven fabrics can be well tuned by adjusting the concentration of PVA/glucose solution and silver ammonia solution. Particularly, the Ag/PVA/PP nonwoven fabric obtained with 30 mM of silver ammonia solution exhibits the best mechanical properties and retains excellent antibacterial activity against *E. coli* even after being washed for 40 cycles, showing promising potential for alleviating the pollution caused by disposable PP nonwoven fabrics. Compared with other literatures, the fabric we obtained via a simpler way exhibits better washability. Furthermore, the as-prepared Ag/PVA/PP nonwoven fabric has desired moisture permeability as well as comfort for wearing, which could be contributive to its application in industry.

## Supplementary Information

Supplementary Figures.

## Data Availability

All data generated or analyzed during this study are included (and its Supplementary Information files).
