# Supplementary material for: Roll to roll in situ preparation of recyclable, washable, antibacterial Ag loaded nonwoven fabric
Source: Sci Rep. 2022 Aug 1;12:13206. doi: 10.1038/s41598-022-17484-6 (PMC9342839; doi:10.1038/s41598-022-17484-6)
Supplement: Supplementary file 1 — Supplementary Figures. [file 41598_2022_17484_MOESM1_ESM.docx]

**Roll to roll in situ preparation of recyclable, washable, antibacterial Ag loaded nonwoven fabric**

Yanfang Xu ^1, 2^, Lulu Tian ^1, 2^, Junfang Li ^1, 2^, Xiaohui Lv ^3^, Fei Li ^1, 2^, Li Sun ^1, 2^, Liyong Niu ^1, 2*^, Xiaohong Li ^1, 2^, Zhijun Zhang ^1, 2*^

1 Engineering Research Center for Nanomaterials, Henan University, Kaifeng, 475004, China

2 Engineering Research Center for Nanomaterials Co. Ltd., Henan University, Jiyuan, 459000, China

3 College of Chemistry and Chemical Engineering, Henan University, Kaifeng, 475004, China

Corresponding Author: Liyong Niu (ly.niu2016@vip.henu.edu.cn)

Zhijun Zhang (zhangzhijun@henu.edu.cn)

Yanfang Xu and Lulu Tian contributed equally to this work.


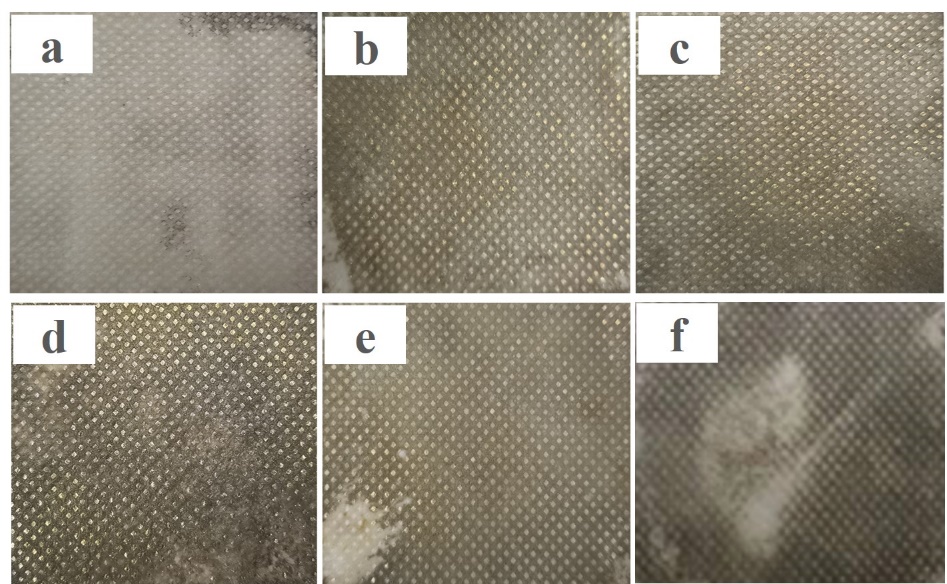


Figure S1. SEM images of Ag/PVA/PP fabrics obtained under the silver ammonia concentration of 50 mM and different molar ratios of PVA/glucose solution to silver ammonia solution (1 : 1 (a), 3 : 1 (b), 5 : 1 (c), 7 : 1 (d), and 9 : 1 (e); f refers to unmodified PP fiber).


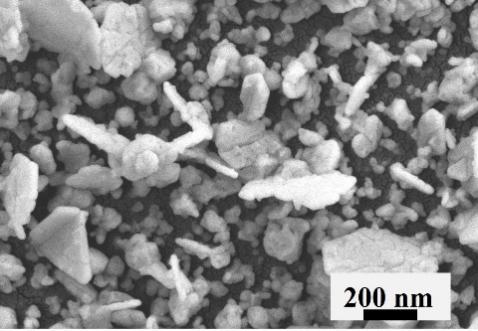


Figure S2. SEM images of Ag/PVA/PP fabrics obtained under the silver ammonia concentration of 30 mM (the molar ratio of glucose to silver ammonia solution is 5:1 and the reaction temperature is 60 ^o^C).


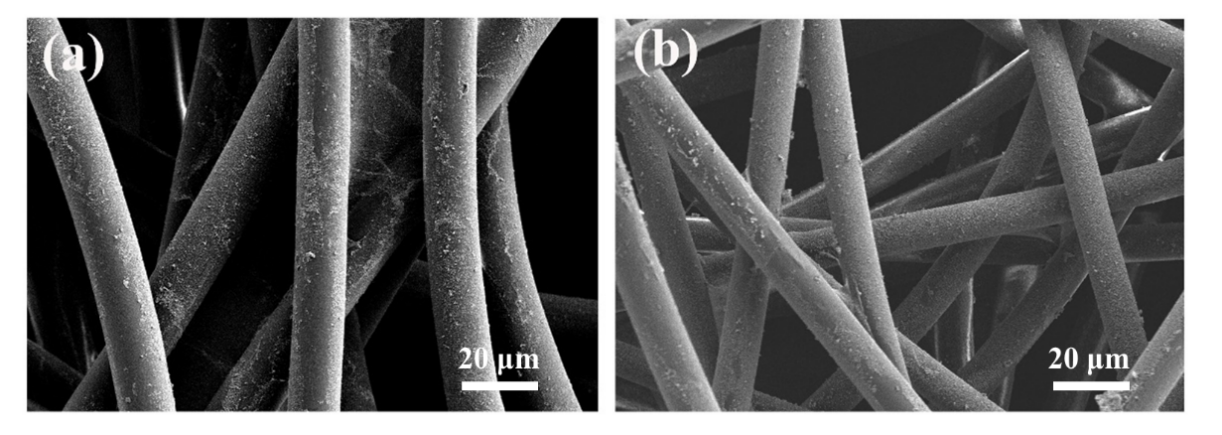


Figure S3. SEM images of Ag/PVA/PP fabrics obtained at (a) 40 ^o^C and (b) 50 ^o^C (the molar ratio of PVA/glucose solution to silver ammonia solution is 5 : 1; and the silver ammonia concentration is 30 mM).





Figure S4. Curve-fitted O 1s spectra of PVA/PP fabric.





Figure S5. Curve-fitted O 1s spectra of Ag/PVA/PP fabric.
